# Alcohol and Cannabinoids Differentially Regulate Macrophage Polarization, with Co-Exposure Producing an Antagonistic Immunomodulatory Effect

**DOI:** 10.3390/ijms27094054

**Published:** 2026-04-30

**Authors:** Esther Penina Shake, Gianelly Vargas Santos, Vijay Sivaraman

**Affiliations:** 1 Department of Biological & Biomedical Sciences, North Carolina Central University, Mary M. Townes Science Building Rm. 2104, 1801 Fayetteville St., Durham, NC 27707, USA; 2 The Julius L. Chambers Biomedical/Biotechnology Research Institute, North Carolina Central University, North Carolina Research Campus, Durham, NC 27707, USA

**Keywords:** crossfading, macrophage polarization, innate immunity, cannabinoid receptors, CB1R, CB2R, alcohol-induced inflammation, alveolar macrophages, immunomodulation

## Abstract

Concurrent alcohol and cannabis use (“crossfading”) is increasingly prevalent, especially among adolescents, yet its toxicological impact on pulmonary innate immunity remains largely unexplored. Alveolar macrophages (AMs) orchestrate inflammatory responses in the lung, and dysregulated macrophage polarization is a hallmark of alcohol-associated lung disease. Although alcohol and cannabinoids individually modulate immune function, the mechanisms by which their co-exposure alters macrophage activation and inflammatory signaling in the lung are largely unknown. AMs are highly sensitive to xenobiotic exposure and play a central role in regulating inflammatory and cytotoxic responses. In this study, we investigated how acute ethanol exposure, synthetic cannabinoid exposure, and their combined exposure affect macrophage viability, polarization, and the release of inflammatory mediators via cannabinoid receptor (CB1R/CB2R)-dependent pathways. Human THP-1-derived macrophages and KG-1 macrophage-like cells were exposed to ethanol, the CB1/CB2 agonist WIN 55,212-2, or both, with selective pharmacological antagonism of CB1R and CB2R. Ethanol exposure activated and polarized macrophages toward a pro-inflammatory M1 phenotype, accompanied by increased secretion of pro-inflammatory cytokines MCP-1, TGF-α, IFN-β, IL-6, and TNF-α. In contrast, WIN 55,212-2 promoted anti-inflammatory M2 polarization and increased IL-10 and IL-4 production. Notably, co-exposure to ethanol and WIN produced an antagonistic immunomodulatory response, characterized by the suppression of ethanol-induced M1 polarization and attenuation of pro-inflammatory cytokine release. Mechanistically, pharmacological CB1R blockade reduced ethanol-induced M1 polarization and cytokine secretion, whereas CB2R blockade exacerbated these effects, underscoring divergent roles for cannabinoid receptors in regulating pulmonary macrophage responses. This study provides novel findings demonstrating the mechanism by which alcohol–cannabinoid co-use reshapes macrophage immune phenotypes and identifies the endocannabinoid system as a potential therapeutic target for alcohol-related inflammatory lung disease.

## 1. Introduction

Alcohol is the third-leading preventable cause of death in the U.S., placing a substantial economic burden; for example, in 2006, alcohol misuse cost Americans over $200 billion. According to the 2023 National Survey on Drug Use and Health (NSDUH), 28.9 million people aged 12 and older (10.2% of this age group) experienced alcohol use disorder (AUD) in the past year. In 2012, alcohol consumption was responsible for 3.3 million deaths worldwide. Alcohol significantly modulates the immune system and alters inflammatory protein expression in both the peripheral and central nervous systems. Chronic and binge alcohol consumption cause complex pulmonary changes and are recognized risk factors for pneumonia [1,2]. Moreover, alcohol abuse increases the incidence of acute respiratory distress syndrome (ARDS) three- to fourfold, a severe lung injury with a 40–50% mortality rate. It also results in poorer clinical outcomes, such as prolonged hospitalization, higher sepsis risk, increased ICU admissions, and greater dependence on mechanical ventilation. Chronic and binge drinking significantly impact the immune system, raising vulnerability to pulmonary infections and worsening inflammatory responses. Despite these associations, the mechanisms underlying alcohol-induced pulmonary inflammation remain unclear [3]. While most alcohol research focuses on the liver, brain, and heart, the lungs remain underexplored despite being particularly vulnerable due to their extensive blood supply and direct ethanol exposure [2,4,5,6]. However, there is a gap in knowledge regarding the adverse effects of alcohol on the pulmonary system and the immune response to this pathogenic factor.

Extensive research shows that smoking marijuana, or cannabis, significantly increases the risk of developing lung diseases due to changes in lung function. Cannabis contains multiple chemicals called cannabinoids (CBs). Cannabinoids comprise phytocannabinoids (e.g., Δ9-THC), synthetic cannabinoids (e.g., WIN 55,212-2), and endocannabinoids like anandamide (AEA) and 2-arachidonoylglycerol (2-AG). Their effects are mainly mediated through cannabinoid receptors CB1R and CB2R, which are part of the endocannabinoid system (ECS). The ECS also involves enzymes that synthesize and degrade endogenous ligands [7,8]. Cannabinoids can modulate the immune system by activating CB1R and CB2R. Most of the well-known effects of cannabis are due to cannabinoids binding to these receptors. Initially, these receptors were identified in the brain [9] Both activate Gi proteins that inhibit adenylate cyclase, preventing ATP from converting to cyclic AMP (cAMP). CB1R is highly expressed in the central nervous system but is also found in several peripheral organs, including the lungs, liver, and kidneys. CB2R is primarily considered the peripheral cannabinoid receptor, present on immune cells, but it is also found in CNS cells. In the lungs, cannabinoid receptors are located on structural cells and most leukocytes [2]. A study examining CB receptor distribution across human organs detected both CB1R and CB2R mRNA in the lungs and bronchial tissue, with the CB1R mRNA levels being significantly higher than CB2R [10]. While most research has focused on the anti-inflammatory role of CB2R, recent preclinical studies suggest that blocking CB1R can reduce inflammation [2]. Notably, exposure to ethanol (EtOH) has also been shown to disrupt the CB system. An increasing amount of compelling evidence indicates that cannabinoid receptors (CB1R and CB2R) play a significant role in alcohol use disorder (AUD). A considerable amount of data link CB1 receptor agonists and antagonists to motivations for alcohol consumption, development of tolerance, dependence, and relapse risk, mostly demonstrated in animal studies [11]. Conversely, this body of evidence primarily focuses on the brain, and few or no studies have examined ethanol’s effects on cannabinoid receptors in the lungs.

Cannabis is the most commonly used drug among individuals with alcohol use disorders (AUDs). It is estimated that 25% of those with AUDs also use cannabis, and 9% are diagnosed with both cannabis use disorders (CUDs) and AUD. After alcohol, marijuana has the highest abuse rate among all drugs [12,13]. Alcohol and cannabis co-use is common, often occurring on the same day, especially among adolescents and young adults. Cannabis use is expected to increase as more states legalize it; by 2018, eight states had legalized recreational use, and 23 states permitted medical marijuana. However, the effects of concurrent alcohol and cannabis use on pulmonary health, particularly innate immunity and airway inflammation, remain poorly understood [14]. A study titled “Alcohol, Cannabis, and Crossfading: Concerns for COVID-19 Disease Severity” describes “crossfading” as using alcohol and cannabis simultaneously, which may synergistically impair lung health by promoting inflammation, worsening pulmonary dysfunction, and increasing susceptibility to respiratory infections like COVID-19 [15,16,17]. Nevertheless, the mechanisms by which alcohol and cannabinoids contribute to pulmonary inflammation remain unclear, highlighting a critical gap in the field.

Alveolar macrophages (AMs), epithelial cells, and dendritic cells form the first line of defense in the lungs, playing a crucial role in maintaining pulmonary homeostasis and initiating immune responses to inhaled pathogens. Among airway immune cells, AMs are by far the most numerous, accounting for over 90% of cells recovered by bronchoalveolar lavage. The primary functions of alveolar macrophages include engulfing dead cells and debris, recycling surfactant, phagocytosing pathogens, coordinating inflammatory processes, and recruiting additional immune cells. When AMs encounter a pathogen, they recognize it via receptors that bind specific molecular motifs on the pathogen. This recognition triggers signaling events that lead to the transcription of pro-inflammatory genes and the release of mediators that recruit leukocytes from the circulation. In a study of human lung-resident macrophages isolated from the lung tissue of patients with adenocarcinoma, CB1R and CB2R mRNA and protein were detected in both tumor-associated and non-tumor-associated macrophages. CB2R levels were higher than those of CB1R, a pattern also observed by the authors in monocyte-derived macrophages. [18].

Macrophages are involved in various pathological processes, including chronic inflammation, tissue remodeling, and tumor growth, due to their ability to produce proinflammatory mediators and angiogenic and lymphangiogenic factors in response to diverse stimuli. Their activation can be broadly divided into two groups: a pro-inflammatory (M1) group, also known as the classically activated group, and an anti-inflammatory (M2) group, also called the alternatively activated group. These groups have different biological functions, cell markers, and secreted cytokines [19,20]. M1 macrophages are proinflammatory and play a central role in host defense against bacterial infections, while M2 macrophages are anti-inflammatory and are associated with the Th2 response, inflammatory inhibition, parasitic infections, tissue remodeling, and tumor progression [18] During inflammation, these two macrophage types are activated alternately, and maintaining a balanced ratio is vital for in vivo homeostasis. M1 macrophages promote inflammation, while M2 macrophages usually suppress it. The phenotype and polarization of macrophages depend on environmental factors [21]. Macrophage polarization is adaptable, meaning that they can shift between M1 and M2 phenotypes [22]. M1 macrophages produce proinflammatory mediators such as IL-6, IL-12, and tumor necrosis factor (TNF), whereas M2 macrophages promote anti-inflammatory responses and help repair damaged tissue [23].

In chronic lung diseases such as COPD and in acute conditions like ARDS, AMs are frequently dysregulated, exhibiting heightened inflammatory profiles or impaired pathogen clearance [24,25]. In COPD, repeated exposure to bacterial products via TLR2 and TLR4 triggers ongoing inflammation, characterized by the release of cytokines such as IL-6, CXCL8, and TNF-α. For years, both the alcohol and pulmonary research communities believed that chronic alcohol use created an anti-inflammatory environment in the lungs. Chronic drinking has been shown to suppress AM activation by reducing the LPS-induced production of pro-inflammatory cytokines such as TNF-α, MIP-2, and CCL3 while increasing the expression of the anti-inflammatory mediator TGF-β. Early studies mostly indicated that chronic ethanol exposure shifts the lung environment toward an anti-inflammatory state by impairing cytokine release from AMs, suggesting that alcohol decreases macrophage activity and weakens immune defenses. However, newer research shows that alcohol exposure can also increase Toll-like receptor (TLR2) levels and elevate IL-8 in bronchoalveolar fluid, implying a pro-inflammatory shift [14]. Additionally, Parker et al. (2024) [2] demonstrated that alcohol enhances CB1/CB2 receptor expression in macrophages and primes the lungs for exaggerated inflammation upon microbial challenge, revealing a dual role for alcohol as both an immunosuppressant and pro-inflammatory modulator, depending on the context. Nevertheless, significant gaps remain in understanding how these effects differ between acute and chronic alcohol exposure, among adolescents versus adults, and how AM functional changes persist over time. Moreover, the long-term immunological effects of cannabis and alcohol co-exposure on AMs are still largely unexplored [2,14]. This study aims to investigate how alcohol and/or cannabinoid (WIN 55,212-2) exposure impacts AM activation and the polarization of AMs into either pro-inflammatory M1 or anti-inflammatory M2 phenotypes. We hypothesize that ethanol promotes pulmonary inflammation by upregulating CB1/CB2 receptors on AMs, thereby polarizing them into the M1 phenotype, characterized by the release of pro-inflammatory cytokines. We also assess whether CB1/CB2 antagonists can mitigate ethanol-induced macrophage polarization and lower the production of pro-inflammatory cytokines and mediators. In our previous lab studies, we demonstrated that adolescent binge alcohol exposure upregulates the expression of cannabinoid receptors 1 and 2 in macrophages and primes the lung for heightened inflammatory responses following microbial challenge. This effect is partly mediated by HMGB-1 signaling and can be reversed with cannabinoid antagonists. Additionally, we found that ethanol and cannabinoid co-exposure worsens pathogen-induced pulmonary inflammation through CB receptor-dependent signaling pathways, providing a mechanistic link between binge alcohol use, endocannabinoid signaling, and immune dysregulation in the lung [2,26]. As a result, this study is based on our previously published in vivo work suggesting a mechanistic role for pulmonary inflammation and aims to elucidate the mechanism underlying that in vivo pathology.

## 2. Results

**Treatment of KG-1 cells with Alcohol and Cannabinoid Causes Toxicity and Cell Death.**

Several studies have reported various mechanisms of alcohol-induced cell death. Ethanol exposure induces macrophage cell death by producing acetaldehyde and high levels of reactive oxygen species during its metabolism. This results in oxidative stress, mitochondrial damage, reduced ATP levels, and the activation of intrinsic apoptotic pathways via cytochrome c release and caspase activation [27,28]. At the same time, ethanol damages membrane integrity and disrupts calcium balance. It also promotes inflammatory responses via TNF-α and DAMPs such as HMGB1, which activate extrinsic apoptotic pathways and further increase macrophage death [1,29]. Some studies have also reported that cannabinoid exposure induces cytotoxicity and apoptosis in various cells [30,31,32]. For this reason, we ran optimization tests to determine the least toxic yet most effective exposure concentrations. KG-1 cells were cultured in the presence and absence of 20 mM EtOH. A second treatment group of cells was cultured in the presence and absence of 20 μM WIN from a 10 mM stock, and the last treatment group received both EtOH and WIN. Cells were exposed to EtOH and WIN for 3 days, with alternating on/off periods, to investigate the acute effects of polysubstance exposure.

**Figure 1:**
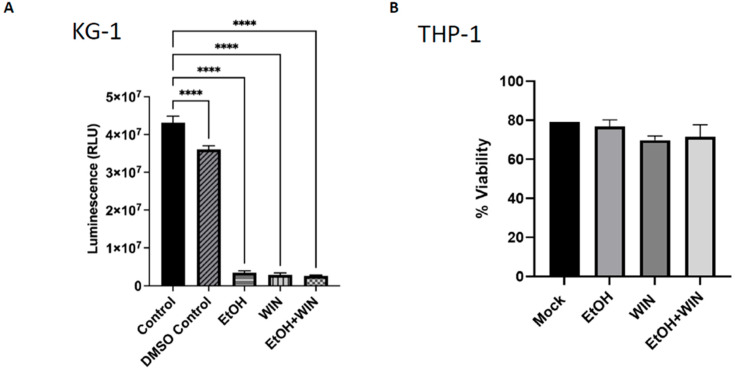
**KG-1 and THP1 Viability Data.** (**A**) Exposure of KG-1 cells to ETOH, WIN, and ETOH + WIN resulted in a diminished luminescent signal due to cell death. KG-1 cells were treated for 3 days, with treatment on and off. Cells were collected after each exposure for Cell Titer Glo analysis. (**B**) THP-1 cells were treated with ETOH, WIN, and ETOH + WIN for 24 h, then analyzed for viability by staining with Fixable Viability Dye eFluor™ 450, which differentiates between live and dead cells. In both the KG-1 and THP-1 cell lines, cell viability was reduced across all treatment groups. **** *p* < 0.0001.

At the endpoint of all experiments, cell viability and toxicity were assessed using Cell Titer Glo (Figure 1A). There is a direct relationship between luminescence (RLU) and the number of viable cells in culture, as measured by ATP quantification from metabolically active cells. The results showed a significant decrease in RLU (*p* < 0.0001) when comparing the control to EtOH, WIN, and EtOH + WIN. There was no significant difference in RLU signal when comparing EtOH to WIN or WIN to EtOH + WIN. The EtOH + WIN treatment group showed a significant decrease (*p* < 0.05) in RLU signal compared with the EtOH treatment group.

Cell viability for THP-1 cells was measured by staining with Fixable Viability Dye eFluor™ 450 (Cat. # 3143127) prior to antibody labeling (Figure 1B). This amine-reactive fluorescent dye, excited by a 405-nm violet laser, penetrates cells with damaged membranes and binds covalently to intracellular proteins, enabling the distinction between live and dead cells during flow cytometry. All treatment groups showed decreased cell viability compared to the untreated group, although the differences were not statistically significant.

The observed difference in cell viability between THP-1 and KG-1 cells might be due to differences in analytical techniques, exposure concentrations, and durations. KG-1 cells showed significantly increased cell death across all treatment groups compared to the controls because they were treated for three days (on alternating days) with ETOH, WIN, and ETOH + WIN, but the WIN concentration was 20 µM, whereas the THP-1 cells were exposed to ETOH, WIN, and ETOH + WIN for only 24 h with WIN at a reduced concentration of 2 µM.

**Alcohol exposure polarizes differentiated THP-1 macrophages and KG-1 cells from an MO state to a pro-inflammatory M1 phenotype**

**Figure 2:**
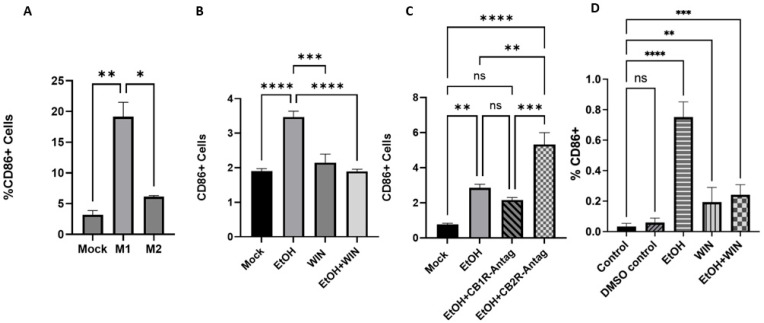
**THP-1 and KG-1 M1 Data.** (**A**–**C**) EtOH exposure polarizes PMA-differentiated THP-1 macrophages from an inactive M0 state to a pro-inflammatory M1 state. M1 phenotype polarization is blocked by CB1R antagonists but increased by CB2R antagonists. (**D**) EtOH exposure polarizes KG-1 macrophages from an inactive M0 state to a pro-inflammatory M1 state. **** *p* < 0.0001, *** *p* < 0.001, ** *p* < 0.01, * *p* < 0.05, ns = not significant.

Our previous in vivo study indicated that binge alcohol exposure modulates the expression of CB1R and CB2R and primes the lung for a more severe inflammatory response to microbial challenge—a response mitigated by cannabinoid antagonists. We examined macrophages in vitro to identify the phenotypic changes driving inflammation and any potential anti-inflammatory effects. We began by exposing KG-1 cells to ethanol. Although the KG-1 cell line is commonly used in immunology research and can differentiate into macrophage-like cells in the presence of phorbol esters, it originates from a bone marrow-derived myeloblastic leukemia and represents an early stage of myeloid maturation. This stage resembles circulating or hematopoietic precursor cells more than tissue-resident macrophages [33]. KG-1 cells primarily reside at the myeloblast/promyelocyte stage and lack the lung-specific environmental imprinting, such as GM-CSF-dependent differentiation, exposure to pulmonary surfactant, and metabolic conditioning, which define alveolar macrophages [34,35,36]. KG-1 cells may reflect characteristics of bone-marrow-derived infiltrating macrophages that populate peripheral tissues such as the skin. Recent studies report that KG-1-derived macrophages tend to be the immature or M0/M2-skewed phenotype, limiting their ability to model the dynamic inflammatory polarization typical of alveolar macrophages [2]. Conversely, THP-1 cells, derived from monocytic leukemia, can be effectively differentiated into mature macrophage-like cells that more closely replicate key functional, metabolic, and inflammatory features of human alveolar macrophages. Many lung-focused studies use PMA-differentiated THP-1 macrophages, which have been widely used to model alveolar macrophage responses to cigarette smoke, airborne particulates, pathogens, and inflammatory stimuli, including cytokine production, phagocytosis, and macrophage–epithelial interactions. These cells show closer alignment with primary alveolar macrophages than other leukemia-based models [32,37,38]. Therefore, we repeated the experiments with THP-1 cells because, although KG-1 cells are useful when a human alveolar macrophage line is unavailable, THP-1-derived macrophages are generally considered a more physiologically relevant and validated in vitro model for studying alveolar macrophage-like immune responses in the lung. Human THP-1 monocytes were differentiated into macrophages by incubation with phorbol 12-myristate 13-acetate (PMA). A 48-h incubation with 20 ng/mL PMA, followed by 24 h in control medium, was chosen as the differentiation protocol. THP-1 macrophages were polarized into M1 macrophages by incubation with 20 ng/mL of IFN-γ and 10 pg/mL of LPS as a positive control (Figure 2A). Differentiated THP-1 macrophages were then treated with or without 0.08% ethanol, which mimics human binge drinking defined as reaching a BAC of 0.08% (0.08 g of alcohol per deciliter of blood) or higher. Cells were treated with a CBR1/2 agonist (2 μM WIN 55,212-2), EtOH + CBR1-Antagonist (2 μM AM281), and EtOH + CBR2-Antagonist (2 μM SR144528) for 24 h at 37 °C in a humidified incubator with 5% CO_2_. Untreated cells served as a negative control. THP-1 cells were stained with fluorescent-tagged antibodies to analyze M1 (APC) and M2 (FITC) surface expression. Fixable Viability Dye (eFluor 450) was used to exclude dead cells (side scatter vs. viability). First, we assessed the polarization of M0 THP-1 macrophages into a pro-inflammatory M1 phenotype (Figure 2B). The highest expression of M1 surface markers was observed in cells treated with EtOH (*p* < 0.0001). Cells treated with the CB1/2 dual agonist showed slightly higher M1 marker (CD86) expression than the mock cells, but the difference was not statistically significant. We further investigated the combined effect of EtOH and WIN on THP-1 macrophages. This combination had an antagonistic effect: EtOH-induced M0 THP-1 polarization to M1 was reduced by co-treatment with EtOH and WIN (*p* < 0.0001). Additionally, we examined the effect of blocking CB1 and CB2 receptors on the surface of M0 THP-1 macrophages prior to EtOH treatment (Figure 2C). The M0 THP-1 cells treated with the EtOH + CB1R antagonists showed reduced M1 macrophage surface marker expression compared to cells treated with only EtOH. Conversely, increased expression of M1 macrophage surface markers was observed when cells were treated with the EtOH + CB2R antagonist compared to the cells treated with EtOH only (*p* < 0.001). Representative flow gating strategy and figures are presented in Supplementary Figure S3.

KG-1 cells were exposed to 20 mM EtOH, a 20 μL aliquot from a 10 mM WIN stock, or both EtOH and WIN for 3 days on and off. Cells were collected and stained 24 h after the final exposure to analyze the surface marker expression induced by polysubstance exposure. KG-1 cells were stained with commercially available fluorescently tagged antibodies to sort for expression of the M1 surface marker (FITC-CD86). eBioscience Fixable Viability Dye (PB450) was used to gate out dead cells from the data. KG-1 cells showed results similar to those of the THP-1 cells. Expression of M1 surface markers was significantly higher across all treatment groups than in the control group (Figure 2D). Interestingly, the EtOH treatment group expressed much higher levels than the WIN and EtOH + WIN treatment groups. This result demonstrates that EtOH may elicit a higher expression of M1 surface markers or that WIN may cause an inhibitory effect on M1 surface marker expression, since there was a significant decrease (*p* < 0.0001) in expression in WIN and EtOH + WIN. No significant difference was observed in M1 expression between the WIN and EtOH + WIN treatment groups. Representative flow gating strategy and figures are presented in Supplementary Figure S1.

**Synthetic cannabinoid receptor dual agonist (WIN 55,212-2) exposure polarizes THP-1 macrophages from an MO state to an anti-inflammatory M2 state. Conversely, in the KG-1 cells, polarization to an anti-inflammatory state was observed across all three treatment groups, with the EtOH + WIN treatment group expressing the highest number of M2 phenotype cells.**

**Figure 3:**
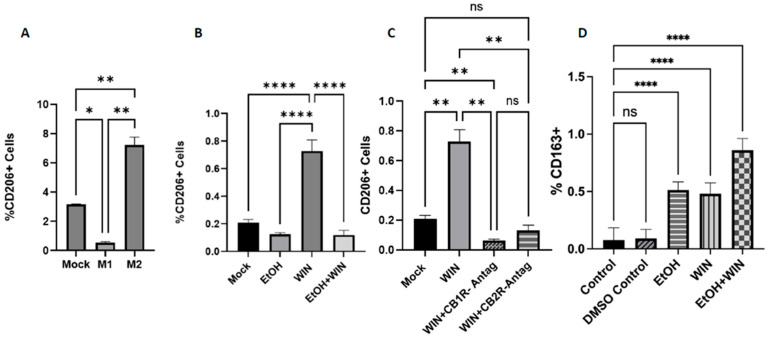
**THP-1 and KG-1 M2 Data.** (**A**–**C**) WIN 55,212-2 exposure polarizes PMA-differentiated THP-1 macrophages from an inactive M0 state to an anti-inflammatory M2 state. M2 polarization is blocked by CB1R and CB2R antagonists. (**D**) KG-1 cells were exposed to EtOH, WIN, and EtOH + WIN for 3 days, with exposure on and off. Cells were collected and stained with fluorescently tagged antibodies to sort for M1 (FITC-CD86) and M2 (CD163-APC). EtOH, WIN, and EtOH + WIN treatments increased anti-inflammatory M2 expression, with the highest expression observed in the EtOH + WIN treatment group. **** *p* < 0.0001, ** *p* < 0.01, * *p* < 0.05, ns = not significant.

Macrophage polarization into alternatively activated macrophages (M2 cells) is induced in vivo and in vitro by IL-4 alone or in combination with IL-13. In this study, we incubated M0 THP-1 macrophages with 20 ng/mL IL-4 for 48 h as a positive control. The M2 phenotype was characterized by measuring the expression of the well-known M2 surface marker CD206 using flow cytometry (Figure 3A). First, we assessed the polarization of M0 THP-1 macrophages toward the anti-inflammatory M2 phenotype (Figure 3B). The highest expression of the M2 surface marker was observed in cells treated with WIN, compared with the untreated group (*p* < 0.0001). Cells treated with EtOH expressed slightly lower levels of the M2 surface marker (CD206) than the mock cells; however, this difference was not statistically significant. We further investigated the effect of co-treatment with EtOH and WIN on M0 THP-1 macrophages. This combination had an antagonistic effect: M0 THP-1 polarization toward an M2 state observed upon exposure to WIN was subsequently reduced by co-treatment with EtOH and WIN (*p* < 0.0001). We further investigated the effect of blocking CB1 and CB2 receptors on the surface of M0 THP-1 macrophages prior to WIN treatment (Figure 3C). M0 THP-1 cells treated with WIN + CB1R antagonists and the WIN + CB2R antagonist showed reduced expression of the M2 surface marker compared to cells treated with WIN (*p* < 0.01). Representative flow gating strategy and figures are presented in Supplementary Figure S4.

KG-1 cells were exposed to 20 mM of EtOH, 20 μM aliquot taken from a 10 mM stock of WIN, and both EtOH and WIN throughout 3 days on and off were collected and stained 24 h after their final exposure to analyze the surface marker expression induced by the polysubstance exposure. KG-1 cells were stained with commercially available fluorescent-tagged antibodies to sort for M2 surface marker expression (CD163-APC). eBioscience Fixable Viability Dye (PB450) was used to exclude dead cells from the analysis. Expression of M2 macrophage surface markers was significantly higher across all treatment groups than in the control group. No significant difference was observed between the treatment groups EtOH and WIN. Treatment group EtOH + WIN expressed significantly higher levels (*p* < 0.0001) of the CD163 surface marker than treatment groups WIN and EtOH (Figure 3D). Representative flow gating strategy and figures are presented in Supplementary Figure S2.

**Acute ethanol and WIN exposure increase the secretion of certain cytokines in differentiated THP-1 and KG-1 cells.**

**Figure 4:**
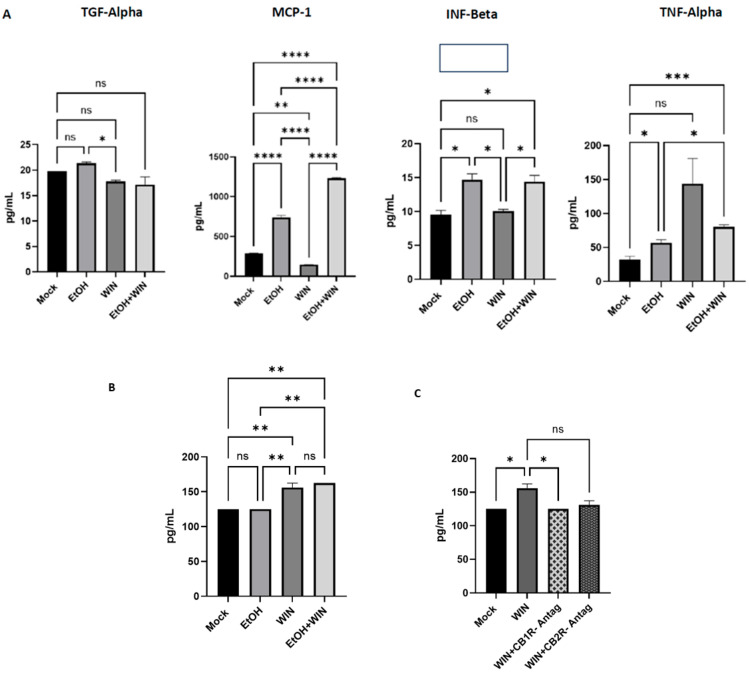
**THP-1 Cytokines.** (**A**) PMA-differentiated THP-1 cells were exposed to EtOH, WIN, and EtOH + WIN for 24 h. The supernatants were then analyzed for TGF-α, MCP-1, IFN-β, and TNF-α expression using the Luminex assay. EtOH treatment significantly upregulated TGF-alpha, MCP-1, and IFN-β expression, with the highest MCP-1 expression observed in EtOH + WIN treated cells. The WIN treatment group had the highest TNF-alpha expression. **THP-1 Cells IL-10 Expression.** (**B**) PMA-differentiated THP-1 cells were exposed to EtOH, WIN, and EtOH + WIN for 24 h. Supernatants were then analyzed for IL-10 expression using the Luminex assay. The WIN and EtOH + WIN treatment groups showed significantly increased IL-10 expression. (**C**) We then blocked CB1/2R with CB1R and CB2R antagonists to determine whether IL-10 expression was mediated by CB1/2R. Pharmacological blockade reduced IL-10 expression. **** *p* < 0.0001, *** *p* < 0.001, ** *p* < 0.01, * *p* < 0.05, ns = not significant.

We then questioned whether cytokines are secreted during ethanol-induced macrophage polarization into the inflammatory M1 state. We collected THP-1 cell supernatant to evaluate protein expression of the following interleukins: TGF-α, INF-Beta, MCP-1, TNF-α, IL-6, and CCL11 (Figure 4A) using Luminex analysis. As expected, the pro-inflammatory chemokine MCP-1 was upregulated in the EtOH-treated cells compared with the mock- and WIN-treated cells (*p* < 0.0001). Surprisingly, the EtOH + WIN treatment group expressed more MCP-1 protein than the EtOH and WIN treatment groups (*p* < 0.0001) and the mock control (*p* < 0.0001).

For TGF-α, the highest expression (in pg/mL) was observed in the EtOH treatment group compared with the WIN treatment group (*p* < 0.05), the EtOH + WIN treatment group, and the mock control (not statistically significant). The TGF-α levels decreased in the WIN and EtOH + WIN treatment groups compared with the mock control group. For IL-6 and CCL11 (commonly known as Eotaxin, a chemokine best known for its role in eosinophil recruitment and TH-2-associated inflammation), although the EtOH treatment group expressed slightly more IL-6 and CCL11 than the WIN and EtOH + WIN treatment groups and the mock control group, the data were not statistically significant.

The highest IFN-β protein expression was observed in the EtOH treatment group compared with the WIN treatment, EtOH + WIN treatment, and mock control groups (*p* < 0.05). Although the EtOH + WIN group had higher IFN-β protein levels than the mock control group (*p* < 0.05), it showed only a slight reduction relative to the EtOH treatment group; this difference was not statistically significant. This suggests that the WIN in this co-treatment exerted little antagonistic effect on IFN-β protein expression compared with the effect on TGF-α expression. TNF-α showed a contrary trend in expression. The WIN treatment group showed the highest TNF-α protein expression (pg/mL) compared with the EtOH treatment and mock control groups. This was an interesting finding, as it contradicted the expression trends observed in the previously mentioned pro-inflammatory cytokines: EtOH-treated groups expressed the highest levels of TGF-α and IL-6, as well as the pro-inflammatory chemokines MCP-1 and CCL11. However, the EtOH and EtOH + WIN treatment groups expressed higher TNF-α protein levels than the mock control group. We also evaluated IL-10 protein expression (Figure 4B). As expected, the WIN treatment group had the highest IL-10 protein levels compared with the EtOH treatment group (*p* < 0.01) and the mock control group (*p* < 0.01). Surprisingly, the EtOH + WIN treatment group expressed slightly higher IL-10 levels than the WIN treatment group, although the difference was not statistically significant. We further assessed whether IL-10 protein expression would be reversed when the CB1R and CB2R receptors were blocked using the CB1R and CB2R receptor antagonists (AM 281 and SR 144528, respectively). A diminished level of IL-10 protein expression was observed in the WIN + CB1R antagonist (*p* < 0.05) and WIN + CB2R antagonist (not statistically significant) pretreatment groups (Figure 4C).

Next, we evaluated the protein expression levels of certain inflammatory cytokines in KG-1 cells. Cell supernatant was collected to measure the inflammatory protein expression of IL-6, IL-4, and TNF-α using ELISA (Supplementary Figure S5). Both EtOH and EtOH + WIN caused a significant increase (*p* < 0.0001) in IL-6 expression. IL-6 expression in WIN also increased significantly (*p* < 0.001). There was a notable decrease in IL-6 production in WIN compared to EtOH, and an increase in IL-6 in the EtOH + WIN group compared to the EtOH group. When comparing IL-6 levels between the EtOH + WIN group and the WIN group, the EtOH + WIN group showed higher IL-6 levels than the WIN group (not statistically significant).

TNFα expression was increased across all the treatment groups compared to the control groups. EtOH expressed an increased level of TNF-α (*p* < 0.05), and exposure to WIN also showed an increased level of TNF-α expression (*p* < 0.001). Surprisingly, the highest TNF-α expression was observed in the WIN treatment group (*p* < 0.0001). IL-4 expression was assessed following the first exposure. All three treatment groups showed increased IL-4 expression compared to the control groups. EtOH treatment resulted in a significant increase in IL-4 expression (*p* < 0.05), but the WIN and EtOH + WIN treatment groups showed a much higher level of IL-4 expression compared to the ETOH and control groups (*p* < 0.0001).

## 3. Discussion

This study employed two cell lines to simulate macrophage responses in both peripheral tissues and the lungs. The KG-1 cell line originates from bone marrow hematopoietic progenitors and represents an early myeloid lineage capable of differentiating into macrophage-like cells upon stimulation. Circulating monocytes produced in the bone marrow migrate into peripheral tissues during inflammation and mature into macrophages that participate in tissue-specific immune functions [39,40]. Therefore, KG-1 cells may mirror the properties of bone marrow-derived infiltrating macrophages that populate peripheral tissues, such as the skin. Macrophages are abundant in skin tissues and play vital roles in inflammatory skin diseases by interacting with keratinocytes, fibroblasts, and other immune cells, underscoring the importance of recruited macrophages in cutaneous immune responses [41]. Conversely, the THP-1 cell line is derived from human monocytic leukemia and represents a monocyte lineage that can be readily differentiated into macrophage-like cells in vitro. Differentiated THP-1 macrophages are commonly used as models of human macrophages and are frequently employed in pulmonary and airway immune research, including studies of macrophage–epithelial interactions and lung inflammation [42]. Thus, KG-1 cells generally reflect the features of bone marrow-derived infiltrating peripheral macrophages, while THP-1-derived macrophages more accurately modeled the differentiated monocyte-derived macrophages used to study pulmonary macrophage responses in vitro.

We aimed to evaluate the effects of alcohol, cannabinoid (WIN 55,212-2), and co-treatment with EtOH and cannabinoid on the activation and polarization of macrophages and subsequent inflammation from 2 different macrophage lineages. In this study, we demonstrate that acute in vitro alcohol exposure activates and polarizes alveolar macrophages from an inactivated M0 state to a pro-inflammatory M1 state, accompanied by the release of pro-inflammatory cytokines. Interestingly, the same effect was observed in both the differentiated THP-1 and KG-1 cells. Conversely, cannabinoid (WIN 55,212-2) exposure activates and skews differentiated THP-1 and KG-1 macrophages toward an anti-inflammatory M2 state, leading to the subsequent release of the anti-inflammatory IL-10 cytokine in THP-1 and IL-4 in KG-1 cells; this effect is reversed in THP-1 cells when we block the CB1R/CB2R using CB1R/CB2R antagonists. However, in KG-1 macrophages, polarization from an inactive M0 state to an anti-inflammatory M2 state was observed across the three treatment groups, with the EtOH + WIN group exhibiting the highest M2 macrophage levels. Interestingly, co-treatment of cells with EtOH and cannabinoids produces an antagonistic immunomodulatory effect, as the alcohol-induced pulmonary inflammation is diminished.

The effects of ethanol on immune cells and alveolar macrophages are paradoxical and highly context dependent, with the literature demonstrating both pro- and anti-inflammatory actions depending on dose, timing, and the presence of secondary inflammatory stimuli. Acute alcohol exposure can transiently enhance innate immune activation, with early increases in circulating leukocytes and augmented LPS-induced TNF-α production, followed within hours by a shift toward an immunosuppressed phenotype characterized by reduced monocyte and NK cell numbers, attenuated IL-1β, and increased IL-10 [43,44]. In contrast, chronic or heavy alcohol exposure consistently impairs host defense, particularly in the lung, by suppressing alveolar macrophage phagocytosis, efferocytosis, and GM-CSF-dependent maturation, thereby predisposing to infection and dysregulated inflammation [3,45,46]. Notably, alveolar macrophages from individuals with alcohol use disorders exhibit a chronically activated, pro-inflammatory mediator profile with elevated TNF-α, CXCL8, and CXCL10, yet simultaneously display functional defects in bacterial clearance, highlighting a state of inflammatory priming coupled with immune paralysis [47]. In our previous lab studies, we showed ethanol-induced modulation of CB1R/CB2R signaling and DAMP release as mechanisms that prime pulmonary immune cells for exaggerated cytokine responses following microbial challenge [2], underscoring that alcohol can be both pro-inflammatory and anti-inflammatory depending on exposure pattern and inflammatory context rather than exerting a uniform immunological effect. However, there is a gap in knowledge on the mechanism behind alcohol-induced pulmonary inflammation, especially the effect of alcohol exposure on the activation of alveolar macrophages that leads to exacerbated pulmonary inflammation. Our data suggest that ethanol exposure activates and polarizes alveolar macrophages from an M0 state to a pro-inflammatory M1 state. In our previous lab studies using KG-1 cells, we demonstrated that EtOH exposure modulated (increased) the expression of both CB1R and CB2R on human macrophages. Interestingly, cells treated with EtOH expressed much higher levels of CB1R than CB2R. Binge ethanol exposure significantly increased pulmonary mRNA expression of the DAMPs HMGB-1 and RAGE, as well as the pro-inflammatory cytokines IL-1β, TNF-α, and MCP-1, indicating lung inflammatory priming. qPCR analysis of mouse lung tissue demonstrated that this ethanol-induced upregulation was significantly attenuated by co-treatment with the CB1 receptor antagonist AM6545, without altering basal gene expression, strongly implicating CB1R signaling as a key mediator of ethanol-dependent pulmonary immune activation [2]. This suggests that ethanol-induced pulmonary inflammation is CB1R/CB2R dependent. In this study, we assessed whether blocking the CB1R/CB2R receptors with cannabinoid receptor antagonists (AM281 and SR144528) could reverse alcohol-induced activation and polarization of THP-1 cells toward a pro-inflammatory M1 state. Interestingly, EtOH + CB1R antagonist treatment diminished M1 state polarization, supporting the point made in our previous study, implicating CB1R signaling as a key mediator of ethanol-dependent pulmonary immune activation; however, EtOH + CB2R treatment increased the polarization of THP-1 cells toward an M1 state. Although ethanol-induced M1 polarization is mediated through cannabinoid receptor signaling, CB1 and CB2 exert opposing immunological functions in monocyte–macrophage cells. CB1 signaling is generally pro-inflammatory, promoting NF-κB/MAPK activation and the expression of M1-associated cytokines and surface markers, such that pharmacologic blockade of CB1 with AM281 removes a major pro-inflammatory driver and diminishes the M1 state. In contrast, CB2 signaling is predominantly anti-inflammatory and immunoregulatory, acting as a compensatory brake that suppresses TLR4–NF-κB signaling and limits excessive macrophage activation; therefore, the inhibition of CB2 with SR144528 removes this negative regulation and consequently exacerbates ethanol-induced M1 polarization. Additionally, we evaluated the cytokine–chemokine profile post-ethanol treatment. EtOH exposure led to the increased production of pro-inflammatory cytokines TGF-alpha, IFN-Beta, TNF-alpha, and MCP-1 in THP-1 cells and IL-6 and TNF-alpha in KG-1 cells.

Although alcohol modulates the immune system and alters the expression of inflammatory proteins in the CNS, the mechanism by which it modulates inflammatory responses in the periphery, particularly the lung, remains unclear. Ethanol can directly affect immune cells, altering cytokine production, cellular activation, and proliferation. Ethanol exposure has been shown to affect the function of macrophages, T cells, and dendritic cells, which are pivotal in immune responses. Ethanol can activate the NF-kB pathway, a crucial transcription factor in the inflammatory response. This can lead to the increased production of pro-inflammatory cytokines such as TNF-α, TGF-α, IFN-β, MCP-1, and IL-6, exacerbating inflammatory responses. In addition, ethanol metabolism produces reactive oxygen species (ROS), which can activate stress-related pathways and cytokine production, further stimulating immune responses [2].

The endocannabinoid system (ECS) includes cannabinoids such as phytocannabinoids (e.g., THC), synthetic compounds (e.g., WIN55,212-2), and endocannabinoids like anandamide (AEA) and 2-arachidonoylglycerol (2-AG). These compounds interact with cannabinoid receptors CB1 and CB2, which are part of the ECS, and involve receptors and enzymes that break down cannabinoids [7,8]. Both plant-derived (e.g., Cannabis sativa) and endogenous (e.g., anandamide [AEA] and 2-arachidonoylglycerol [2-AG]) types have demonstrated immunomodulatory and anti-inflammatory effects in various disease models, including acute lung injury and asthma in C57BL/6 mice. Our data suggest that the synthetic cannabinoid receptor agonist (WIN55,212-2) activates and polarizes THP-1 and KG-1 macrophages from an inactive M0 state to an anti-inflammatory M2 state. There is also the upregulation and release of anti-inflammatory cytokines IL-10 and IL-4. The pharmacologic blockade of both CB1R and CB2R reduced polarization of M0-differentiated THP-1 macrophages toward an anti-inflammatory M2 state and IL-10 secretion, demonstrating that signaling through both receptors contributes to the synthetic cannabinoid’s anti-inflammatory effect. Surprisingly, in differentiated THP-1 macrophages, although TNF-α secretion increased with EtOH treatment, WIN 55,212-2 treatment produced the highest TNF-α levels. In contrast, KG-1 macrophages expressed higher TNF-α protein across the three treatment groups, with the highest expression observed in the ETOH + WIN treatment group. A study by Cohen et al., 2023 [48] also reported that THC exerted the most effective anti-inflammatory effect at lower concentrations in both J774A1 macrophages and EGCs compared to other tested phytocannabinoids. In that regard, TNF-α secretion was significantly reduced at low THC concentrations (0.05–0.5 µg/mL); however, at higher THC concentrations (above 0.55 µg/mL), TNF-α secretion increased, indicating a biphasic effect [48].

Our study shows that co-exposure to EtOH and the synthetic cannabinoid agonist WIN 55,212-2 had an antagonistic immunomodulatory effect. We report a novel finding that in both differentiated THP-1 and KG-1 macrophages, the M1-polarized macrophage state was reduced in the EtOH + WIN co-treatment group. These results suggest that WIN 55,212-2’s immunoregulatory effect reduced alcohol-induced inflammation. Although extensive research has clarified the effects of alcohol and cannabinoids on the liver, brain, and cardiovascular system, the lungs remain underexplored despite direct exposure to circulating ethanol, cannabinoids, and their metabolites. In this study, we demonstrated the effect of co-treatment with these two substances in the lung. This information is very important, especially for therapeutic interventions against alcohol-induced inflammatory lung diseases.

It is important to recognize some limitations of our study. While we used the human KG-1 and THP-1 cell lines as in vitro models of cannabinoid receptor signaling in macrophages and of inflammatory responses similar to those of alveolar macrophages, they cannot fully replace primary human alveolar macrophages. KG-1 cells are commonly used in immunology research and are derived from a patient with erythroleukemia who progressed to acute myelogenous leukemia. These cells are mainly myeloblasts or promyelocytes that can differentiate into macrophage-like cells in the presence of phorbol esters [49]. Although recent research suggests that KG-1-derived macrophages exhibit an M0 or M2-like phenotype, their leukemic progenitor origin and immature hematopoietic stage limit their relevance as models for tissue-resident alveolar macrophages [2], which develop from fetal monocytes and undergo lung-specific differentiation driven by GM-CSF and surfactant exposure [34,35]. Conversely, THP-1 cells are derived from a human acute monocytic leukemia and can efficiently differentiate into mature macrophage-like cells with defined inflammatory and polarizing responses [50,51]. Importantly, THP-1-derived macrophages have been widely used as an in vitro model of alveolar macrophage-like inflammatory signaling, especially in studies of pulmonary innate immunity, ethanol exposure, cigarette smoke, and particulate matter [52,53]. However, a study by Tedesco et al., 2018, indicated that although THP-1-derived macrophages are useful as a simplified model for studying human macrophage processes like polarization and its functional implications, they are not ideal substitutes in comprehensive immunopharmacology and drug screening, because they do not fully replicate the specific transcriptional, metabolic, and immunoregulatory features of primary human alveolar macrophages [38]. Despite these limitations and the lack of a commercially available human alveolar macrophage cell line, using both KG-1 and THP-1 cells provided complementary insights into macrophage cannabinoid receptor signaling, with THP-1 cells serving as a more biologically relevant model for in vitro studies of alveolar macrophage activation and polarization.

Our experiments primarily utilized a synthetic cannabinoid receptor agonist, WIN 55,212-2. In everyday use, humans consume cannabis through methods like smoking, vaping, or eating edibles. The effects of isolated cannabinoid agonists may not fully represent how cannabis affects the immune system, lungs, or brain in its natural form. Although THC would have been preferable, we chose WIN 55,212-2 because it is commercially available and commonly used in cannabinoid research across many laboratories. While not approved for human clinical use, WIN 55,212-2 is widely employed in preclinical studies to investigate the endocannabinoid system and CB1/CB2 signaling pathways. Unlike natural cannabinoids such as THC or CBD, WIN 55,212-2 is synthetic and has a unique chemical structure, making it a valuable pharmacological tool for studying macrophage polarization and CB receptor signaling in vitro, as demonstrated by our work and others. The CB-agonists and antagonists used in our study were soluble in carriers such as DMSO, DMF, or ethanol, facilitating cell treatment. In contrast, whole cannabis requires combustion, vaporization, or extraction, which introduces variability in administration and absorption [26]. For future studies, we will be performing aerosolized cannabinoid exposures on the air–liquid interface (ALI).

In our previous lab studies, we demonstrated that EtOH modulates the expression of cannabinoid receptors 1 and 2 in lung macrophages. We reported a novel finding: EtOH activates and primes lung macrophages to elicit a more severe inflammatory response upon microbial pathogen challenge, and this response is mediated by CBRs. Additionally, we investigated the effects of binge cannabinoid exposure on pathogen-induced pulmonary inflammation using a binge ethanol + cannabinoid adolescent mouse model of Klebsiella pneumoniae (K. pneumoniae) infection. We reported that adolescent cannabinoid exposure primes the lung to a more severe inflammation in adulthood upon subsequent microbial challenge, and this response was mitigated by cannabinoid antagonists. We showed that ethanol and cannabinoid preexposure followed by microbial challenge yielded CBR-dependent pulmonary immune activation via danger-associated molecular pattern (HMGB1 protein) release. There is a knowledge gap regarding whether the HMGB1 protein is released by accumulated apoptotic cells or by activated AMs. For this study, we investigated how EtOH and cannabinoids interact mechanistically with alveolar macrophages in the lung, leading to AM activation, polarization, and the upregulation of proinflammatory cytokines. We also assessed whether the co-exposure of EtOH and cannabinoid (WIN 55,212-2) would have an immunoregulatory effect that would reduce the alcohol-induced pulmonary inflammation. In this study, we demonstrated that acute binge exposure to cannabinoids and alcohol exerts opposite inflammatory effects, as shown by the activation and polarization of AMs and the cytokine array profiles observed in vitro using the THP-1 and KG-1 cell lines (Figure 2, Figure 3 and Figure 4). Our in vitro study using THP-1 and KG-1 macrophages suggests that acute binge ethanol exposure activates and polarizes AMs to a pro-inflammatory M1 state with the subsequent upregulation of proinflammatory cytokines TGF-α, IFN-β, TNF-α, and MCP-1, and this effect is reversed in differentiated THP-1 cells by the pharmacological blockade of CB1R and CB2R using CB antagonists (AM281 and SR144528). On the contrary, cannabinoid exposure activates and skews AMs toward an anti-inflammatory M2 state in differentiated THP-1 macrophages, leading to the release of the anti-inflammatory IL-10 cytokine. In contrast, in KG-1 macrophages, the polarized M2 state was observed in all three treatment groups, with the EtOH + WIN treatment group expressing the highest levels of anti-inflammatory M2 macrophages. Co-exposure to EtOH and cannabinoids exerts an immunomodulatory effect, reducing alcohol-induced inflammation. These studies are the first (to our knowledge) to demonstrate CBR-dependent macrophage activation status and cytokine secretion using human cells. Future studies will evaluate primary human cells for mechanistic insight. Further research is needed to determine whether AM activation results from direct systemic ethanol exposure or from an indirect effect mediated by alcohol-induced gut-derived endocannabinoids that circulate to the lungs and activate AMs. This distinction is essential for understanding the mechanisms underlying alcohol-related lung immune dysfunction and could help pinpoint novel gut–lung axis targets for interventions.

## 4. Materials and Methods

**THP-1 cells culture, differentiation, and treatment**

Human monocytic THP-1 cells were maintained in culture in Roswell Park Memorial Institute medium (RPMI 1640 medium, Invitrogen, Carlsbad, CA, USA) containing 10% heat-inactivated fetal bovine serum (Invitrogen, Carlsbad, CA, USA) and supplemented with 10 mM antibiotic (mention the name) and 50 pM β-mercaptoethanol. THP-1 monocytes were then seeded into 24-well plates at a density of 5.0 × 105 cells/mL and cultured in maintenance media (RPMI medium) supplemented with 20 ng/mL phorbol 12-myristate 13-acetate (PMA; Sigma-Aldrich, St. Louis, MO, USA) for 48 h to differentiate them into macrophages (THP-1-derived macrophages). The cells were then left to rest and recover in maintenance media for 24 h, free from PMA. Macrophages were polarized into M1 macrophages (positive control) by incubation with 20 ng/mL of IFN-γ (R&D Systems, Minneapolis, MN, USA, #285-IF) and 10 pg/mL of LPS (Sigma-Aldrich, St. Louis, MO, USA, #L6529). Macrophage M2 polarization (positive control) was obtained by incubation with 20 ng/mL of interleukin-4 (R&D Systems, Minneapolis, MN, USA, #204-IL/CF). We conducted dose–response tests using a dose–response curve to determine the optimal ethanol and WIN 55,212-2 concentrations that elicit an immunological response without significant cytotoxicity (Supplementary Figure S6). Differentiated THP-1 macrophages were then treated in the presence or absence of 0.08% ethanol for 24 h at 37 °C in a humidified incubator in 5% CO_2_. The 0.08% ethanol concentration mimics binge drinking, which is defined as reaching a BAC of 0.08% (0.08 g of alcohol per deciliter of blood) or higher. Other studies have also used this percentage [2]. A subset of cells was treated with or without WIN, AM, and SR (each at 2 μM).

**KG-1 cell culture and treatment**

The KG-1 cell line used in the experiments was obtained from the American Type Culture Collection (ATCC, Cat. No. CRL-8031). The human KG-1 suspension cell line is derived from the bone marrow of a patient with erythroleukemia who subsequently developed acute myelogenous leukemia. The cell line was cultured in Iscove’s modified Dulbecco’s medium (IMDM) supplemented with 20% fetal bovine serum (FBS). Cultures were incubated in a CO_2_-humidified atmosphere at 37 °C and maintained at a cell density of 2 × 10^5^–1 × 10^6^ cells/mL. KG-1 cells were plated at 500,000 cells per well in a 12-well plate for exposure. There were five experimental groups: the control group consisting of KG-1 cells in medium, the dimethyl sulfoxide (DMSO) vehicle control group, the ethanol (EtOH) group, the synthetic cannabinoid WIN 55,212 (WIN) group, and the EtOH and WIN group. The DMSO control group received 5 μM DMSO. The rationale for including a DMSO control group was to account for the DMSO used to reconstitute WIN. The EtOH treatment concentration was 20 mM, and the WIN treatment concentration was 20 μM of a 10 mM stock. The exposure schedule was 3 days on and off. On the first day, cells were exposed to EtOH with or without WIN. The second day was a rest day, and cells were not exposed to any treatment. On the second day, cell viability was assessed to evaluate the effects of exposure 1, and cell supernatants were collected for ELISA. On the third day, cells were given a second exposure to EtOH and WIN. On the fourth day, cells and supernatants were collected for flow cytometry analysis and ELISA experiments. Cell viability was measured to analyze the effects of exposure 2. Cytotoxicity and viability were evaluated by measuring the luminescent signal from KG-1 cells 24 h after the first exposure and again 24 h after the second exposure.

**Cannabinoid compounds used in the study**

CBR1/2 dual agonist

WIN (WIN 55,212-2; [(3R)-2,3-dihydro-5-methyl-3-(4-morpholinylmethyl) pyrrolo [1,2,3-de]-1,4-benzoxazin-6-yl]-1-naphthalenyl-methanone, monomethanesulfonate mesylate) was purchased from Cayman Chemical (Item # 10009023), and stock solutions were prepared according to the manufacturer’s protocols. Briefly, WIN 55,212-2 (mesylate) is supplied as a crystalline solid. A stock solution was prepared by dissolving WIN 55,212-2 in DMF (30 mg/mL) and then diluting it with PBS at a 1:4 DMF-to-PBS ratio (pH 7.2). WIN 55,212-2 is a synthetic agonist of cannabinoid receptors that mimics endogenous endocannabinoids by activating both CB1 and CB2 receptors, thereby regulating neurotransmission, immune function, inflammatory responses, and downstream intracellular signaling pathways [2].

CB1R antagonist:

CB1R, AM281 (1-(2,4-dichlorophenyl)-5-(4-iodophenyl)-4-methyl-N-4-morpholinyl-1H-pyrazole-3-carboxamide) was purchased from Cayman Chemical (Item # 10006972) and stock solutions were prepared following the manufacturer’s protocols. Briefly, AM281 is supplied as a crystalline solid. A stock solution was prepared by dissolving AM281 in DMF (1 mg/mL) and then diluting it 1:5 in PBS (pH 7.2).

CB2R antagonist:

SR144528 5-(4-chloro-3-methylphenyl)-1-[(4-methylphenyl)methyl]-N-[(1S,2S,4R)-1,3,3-trimethylbicyclo [2.2.1]hept-2-yl]-1H-pyrazole-3-carboxamide) was purchased from Cayman Chemical (Item# 9000491) and stock solutions were prepared following the manufacturer’s protocols. Briefly, SR 144,528 is supplied as a crystalline solid. A stock solution was prepared by dissolving SR 144,528 in ethanol (30 mg/mL) and then diluted in PBS to a 1:1 ethanol:PBS (pH 7.2) solution.

**Flow cytometry analysis**

Flow cytometry was used to distinguish between M1 and M2 macrophage activation from the initial M0 state. THP-1 and KG-1 macrophages were stained with fluorescently tagged antibodies to sort for surface markers of the proinflammatory M1 phenotype (Human classical activated macrophages CD86 APC-conjugated antibody, Cat. # 555660) and the anti-inflammatory M2 phenotype (PerCP-Cy™5.5 Mouse Anti-Human CD206, Cat. # 551,136 or a CD163 monoclonal antibody conjugated to FITC) following the manufacturer’s instructions. Fixable Viability Dye eFluor 450 (Cat. # 3143127) was used to gate out dead cells by side scatter vs. viability. Data acquisition was performed using a CytoFLEX flow cytometer (Beckman Coulter, Miami, FL, USA). The CytExpert acquisition software (version 2.0;Beckman Coulter, Miami, FL, USA) was used to acquire samples, and FCS Express (version 6; DeNovo Software, Pasadena, CA, USA) was used for analysis. For each sample, at least 20,000 events were collected. Representative flow gating figures are presented in Supplementary Figures S1–S4.

**Luminex analysis**

Cytokine concentrations in the THP-1 cell culture supernatants were quantified using a multiplex bead-based Luminex assay according to the manufacturer’s protocol. Briefly, the cell culture supernatant samples were diluted 2-fold in the calibrator diluent before analysis. Samples, standards, and controls were assayed in duplicate using the Human XL Cytokine Premixed Kit (R&D Systems, Minneapolis, MN, USA) and analyzed on a Luminex 200 system. A total of 50 μL of diluted sample, standard, or control was added to each well, followed by 50 μL of antibody-immobilized magnetic microparticles. Plates were incubated for 2 h at room temperature with agitation, washed three times, and incubated sequentially with biotinylated detection antibodies (1 h) and streptavidin–phycoerythrin (30 min). After final washes, beads were resuspended in wash buffer and acquired on the Luminex 200 system with a minimum of 50 beads per analyte per well. Cytokine concentrations were calculated from standard curves generated using five-parameter logistic regression, and values were corrected for sample dilution.

**Enzyme-linked immunosorbent assay**

The KG-1 cell supernatant from each sample was collected after each exposure and analyzed to determine cytokine concentrations using BD OPTEIA™ Human ELISA sets for interleukin-6 (IL-6), interleukin-4 (IL-4), and tumor necrosis factor (TNF). Each ELISA followed the same protocol, using the respective antibodies for IL-6, IL-4, and TNF. Briefly, 96-well ELISA plates were coated with 100 μL of a 1:250 dilution of the capture antibody and incubated overnight at 4 °C. The following day, wells were aspirated and washed 5 times with 300 μL of Wash Buffer. Plates were then blocked with 200 μL of Assay Diluent and incubated at room temperature for 1 h. After the 1-h incubation, plates were aspirated and washed 5 times with 300 μL of Wash Buffer. Then, 100 μL of each standard, sample, and control was added to the appropriate wells, and the plate was sealed and incubated at room temperature for 2 h. After 2 h of incubation, plates were aspirated and washed again. Subsequently, 100 μL of Working Detector was added to each well and incubated at room temperature for 1 h. Next, the plate was aspirated and washed 10 times. Following the final wash step, 100 μL of substrate solution was added to each well and incubated for 30 min at room temperature in the dark. Finally, 50 μL of stop solution (1 M phosphoric acid) was added, and absorbance was read at 450 nm within 30 min of stopping the reaction.

**Statistical analysis**

All data are presented as the mean ± standard error of the mean (SEM). In studies with more than two groups, statistical analyses were performed using one-way analysis of variance (ANOVA) followed by a Tukey–Kramer post hoc test to detect differences between individual experimental groups (GraphPad Prism version 10, San Diego, CA, USA). If the data were not normally distributed, nonparametric statistical analysis using the Kruskal–Wallis test was performed. Values of *p* < 0.05 were considered statistically significant.

## 5. Conclusions

This study demonstrates that ethanol and cannabinoids exert opposing effects on macrophage polarization and inflammatory signaling, with significant differences across macrophage models. Ethanol consistently promoted a pro-inflammatory M1 phenotype, leading to increased secretion of cytokines and chemokines, including MCP-1, TNF-α, TGF-α, IFN-β, and IL-6. In contrast, the synthetic cannabinoid agonist WIN 55,212-2 promoted an anti-inflammatory M2 phenotype characterized by elevated production of IL-10 and IL-4. Notably, co-exposure to ethanol and cannabinoids produced an antagonistic immunomodulatory effect, in which cannabinoid signaling attenuated ethanol-induced M1 polarization and decreased pro-inflammatory cytokine release, demonstrating an active interplay between these pathways.

These findings build directly on our previously published in vivo work, which demonstrates that ethanol exposure induces pulmonary inflammation and primes the lung for exaggerated immune responses in a cannabinoid receptor (CB1R/CB2R)-dependent manner. In that study, ethanol was shown to modulate CB receptor expression, promote DAMP release and pro-inflammatory cytokine production, and when CB1R and CB2R were pharmacologically blocked, attenuate ethanol-induced immune activation, establishing a mechanistic link between alcohol-induced inflammation and cannabinoid receptor signaling [2]. Consistent with these observations, the current study confirms and extends these findings at the cellular level by demonstrating that ethanol drives macrophage polarization toward a pro-inflammatory M1 phenotype via CB receptor-dependent pathways, whereas cannabinoid signaling counter-regulates this response. Together, these data provide mechanistic insight into the macrophage-specific processes underlying the pulmonary inflammatory phenotype observed in vivo.

Despite these conserved effects, key similarities and contradictions were observed between THP-1-derived macrophages and KG-1 cells. In both models, ethanol enhanced pro-inflammatory signaling, and cannabinoid exposure promoted anti-inflammatory responses, supporting a shared biological response across macrophage populations. However, THP-1 macrophages exhibited more defined, receptor-dependent polarization patterns, whereas KG-1 cells displayed broader, less tightly regulated responses, including a tendency toward M2-like polarization across multiple treatment conditions and variable cytokine profiles. These differences likely reflect the distinct biological origins of the models, with THP-1 cells representing differentiated monocyte-derived macrophages commonly used to model pulmonary macrophage-like responses, and KG-1 cells representing bone marrow-derived, immature myeloid cells that resemble circulating or peripheral infiltrating macrophage precursors.

Collectively, these findings indicate that although the opposing immunological effects of ethanol and cannabinoids are conserved, the magnitude and regulation of these responses depend on macrophage lineage and maturation state. This study provides a mechanistic bridge between in vivo pulmonary inflammation and in vitro macrophage biology, highlighting the endocannabinoid system, particularly the divergent roles of CB1R and CB2R, as a key regulator of alcohol-induced immune dysregulation. These insights have important implications for understanding the immunological consequences of alcohol–cannabinoid co-use and support the potential of targeting cannabinoid receptor signaling as a therapeutic strategy for alcohol-associated inflammatory diseases. Although our study sheds light on the mechanistic pathway for alcohol-induced pulmonary inflammation and the immunomodulatory effects of cannabinoids, further studies are needed, and substantial evidence must be acquired before these novel cannabinoid receptor pathway-targeted therapeutic approaches can be used in the clinic to treat alcohol use disorders.

## Data Availability

The original contributions presented in this study are included in the article/Supplementary Materials. Further inquiries can be directed to the corresponding author.

## Supplementary Materials

The following supporting information can be downloaded at: https://www.mdpi.com/article/10.3390/ijms27094054/s1.
