# Supplementary material for: Alcohol and Cannabinoids Differentially Regulate Macrophage Polarization, with Co-Exposure Producing an Antagonistic Immunomodulatory Effect
Source: Int J Mol Sci. 2026 Apr 30;27(9):4054. doi: 10.3390/ijms27094054 (PMC13163345; doi:10.3390/ijms27094054)
Supplement: Supplementary file 1 [file ijms-27-04054-s001.zip › ijms-4237546-supplementary.pdf]

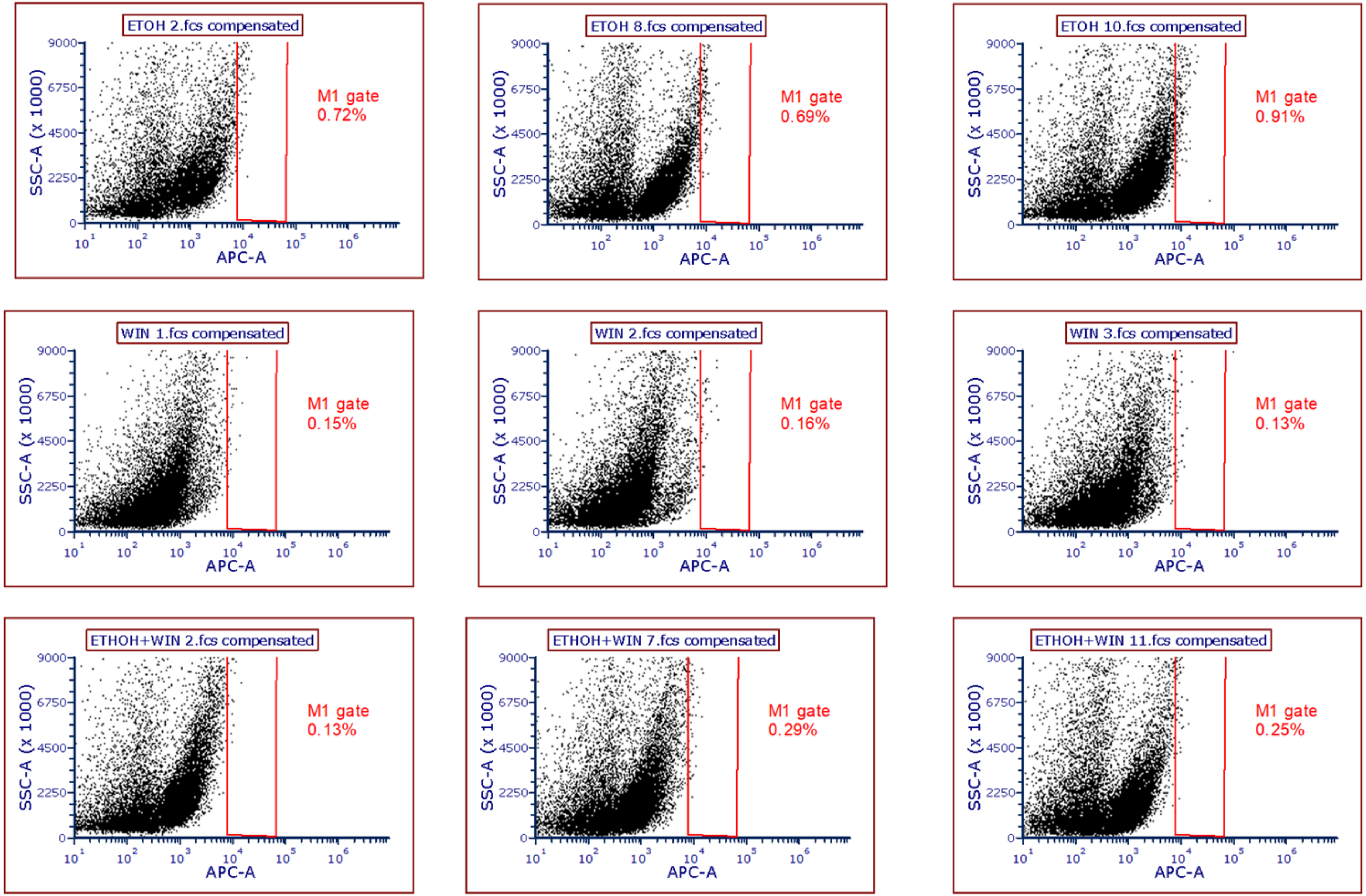

Figure S1. KG-1 Gating M1

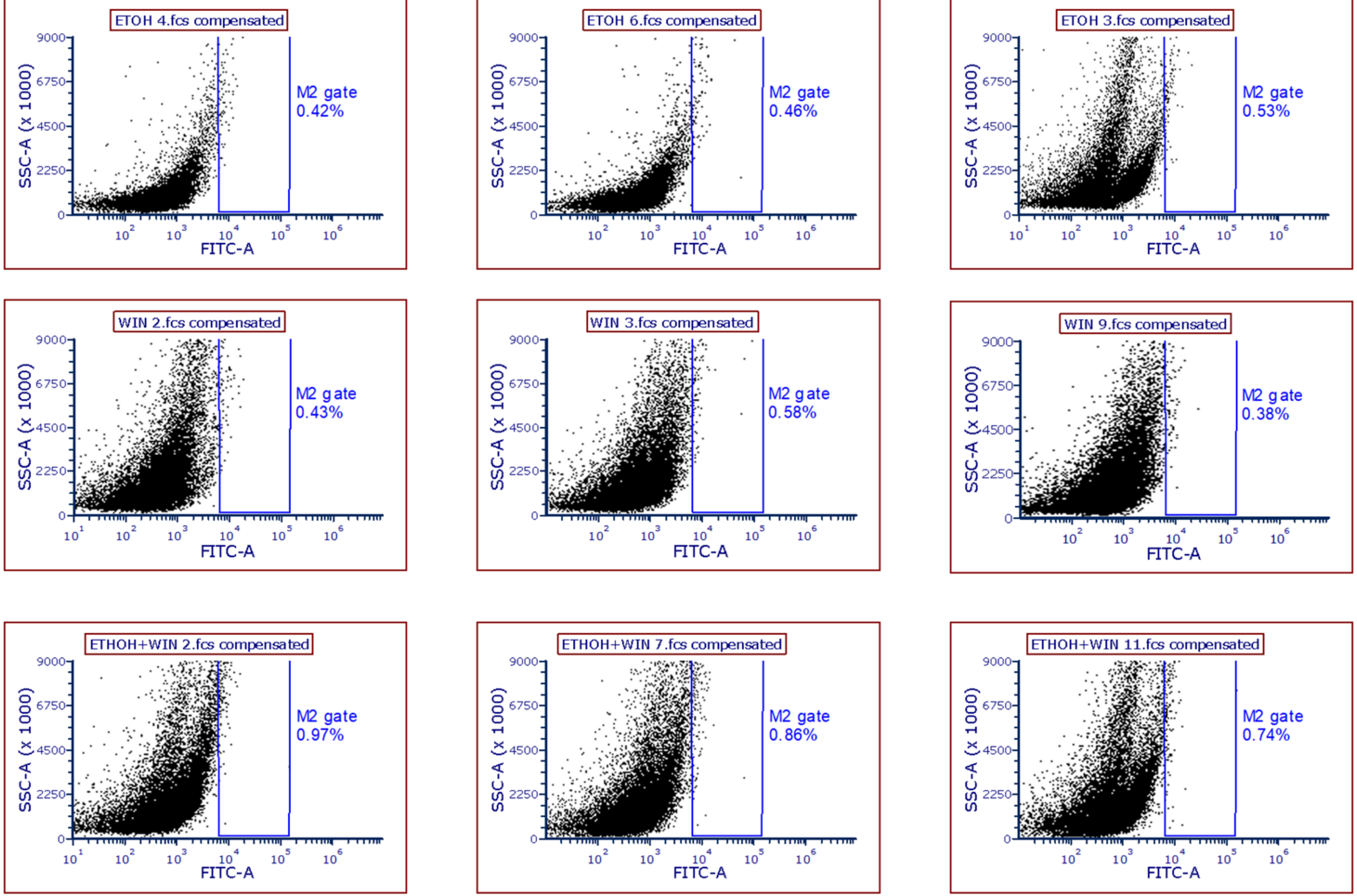

Figure S2. KG-1 Gating M2

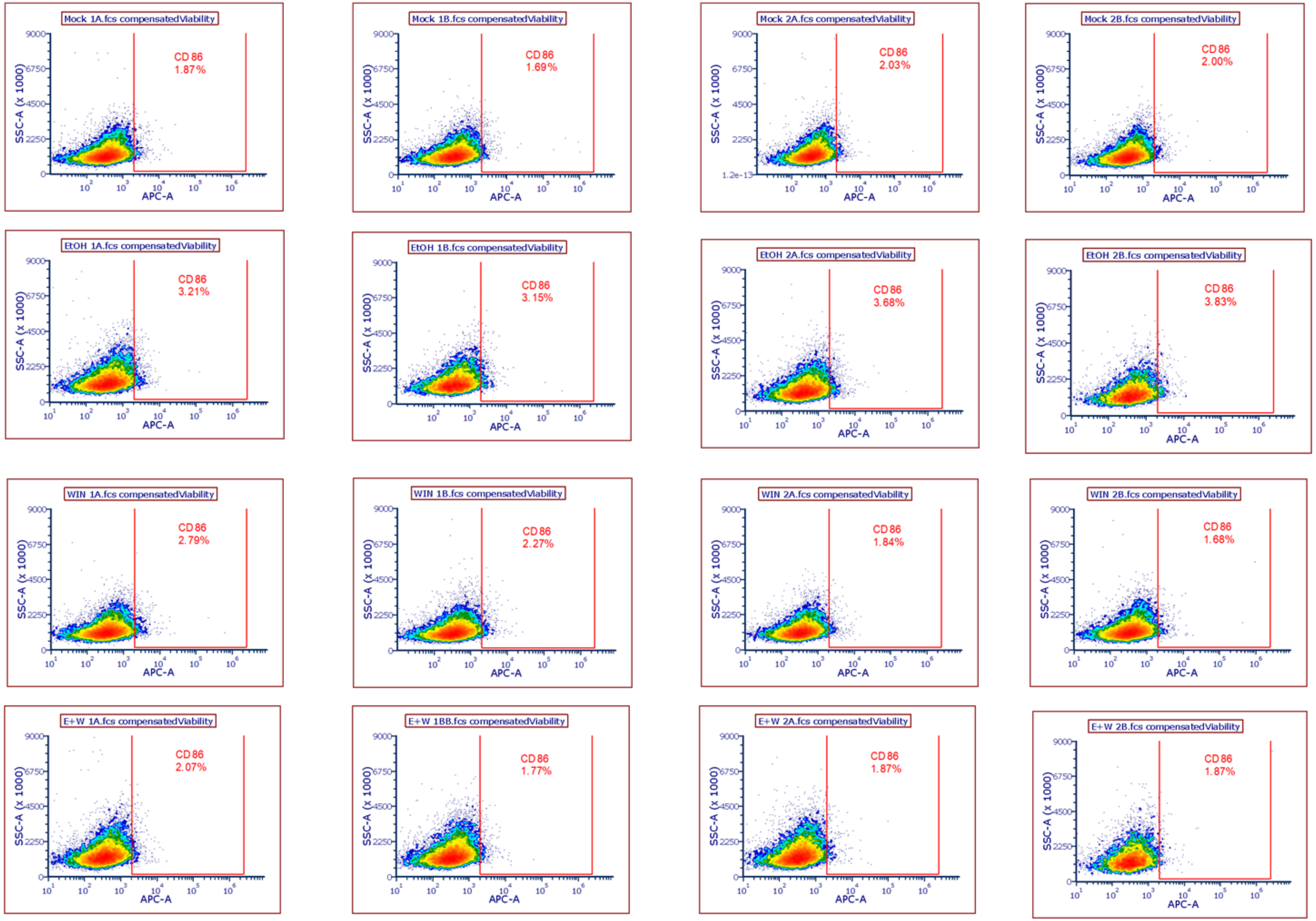

Figure S3. THP-1 Gating M1

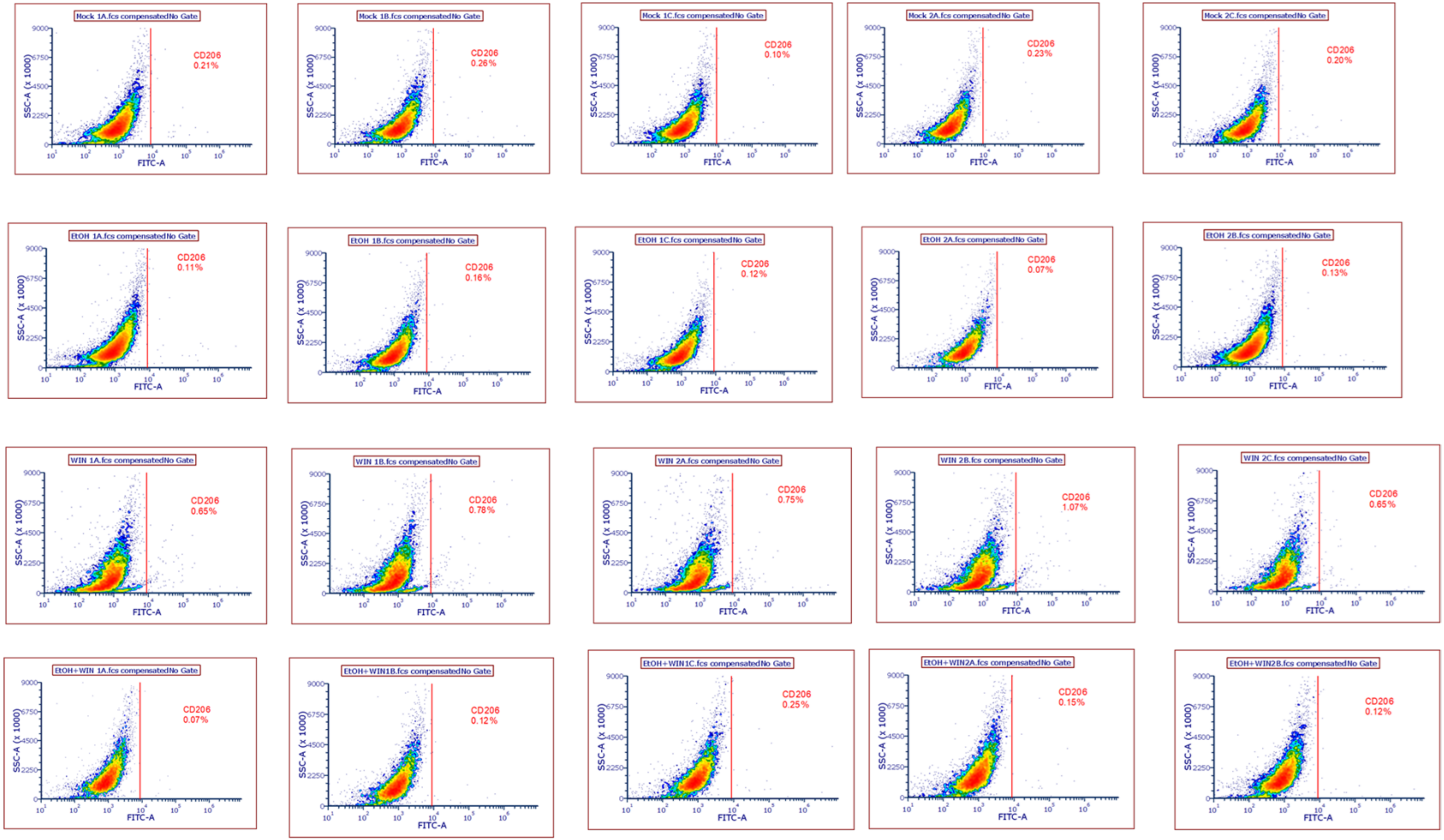

Figure S4. THP-1 Gating M2

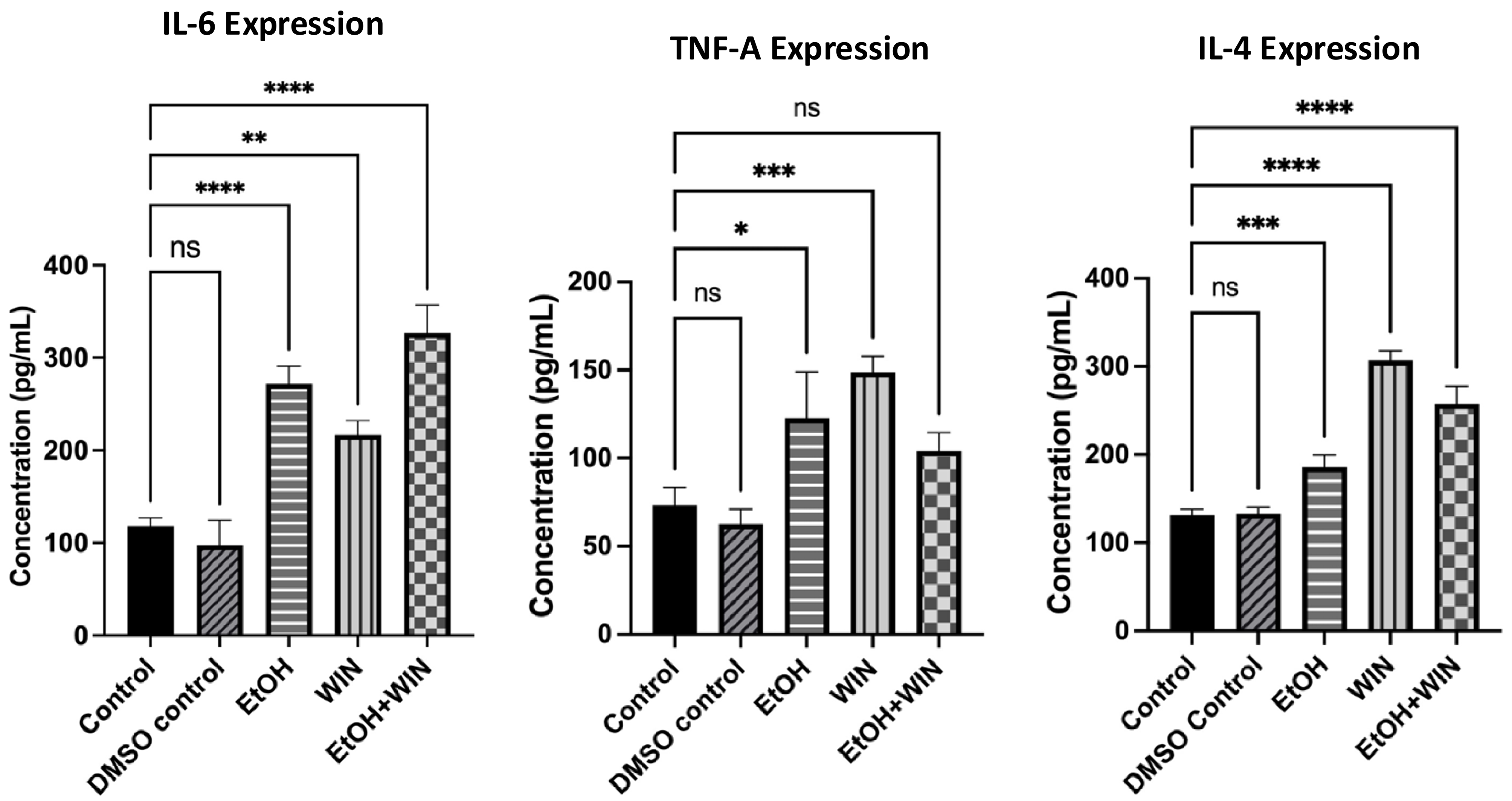

Figure S5. KG-1 Cytokines

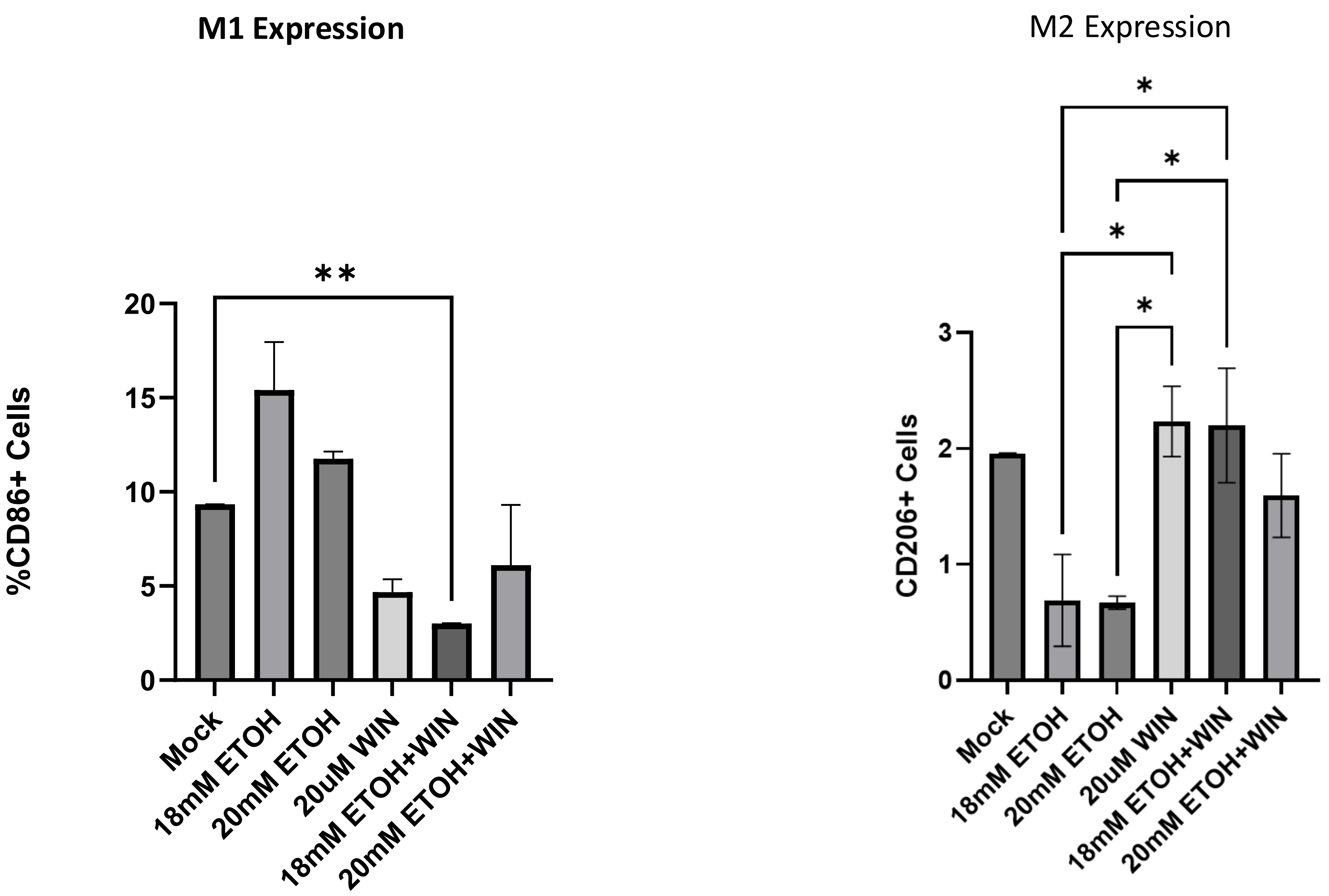

Figure S6. THP-1 M1 and M2 Expression Preliminary Work
